# Acne, Microbiome, and Probiotics: The Gut–Skin Axis

**DOI:** 10.3390/microorganisms10071303

**Published:** 2022-06-27

**Authors:** Pedro Sánchez-Pellicer, Laura Navarro-Moratalla, Eva Núñez-Delegido, Beatriz Ruzafa-Costas, Juan Agüera-Santos, Vicente Navarro-López

**Affiliations:** 1 MiBioPath Research Group, Department of Clinical Medicine, Health Sciences Faculty, Catholic University of Murcia, Campus de los Jerónimos 135, 30107 Murcia, Spain; pedro.sanchez@bioithas.com (P.S.-P.); laura.navarro@bioithas.com (L.N.-M.); eva.nunez@bioithas.com (E.N.-D.); beatriz.ruzafa@bioithas.com (B.R.-C.); juan.aguera@bioithas.com (J.A.-S.); 2 Infectious Diseases Unit, University Hospital of Vinalopó, Carrer Tonico Sansano Mora 14, 03293 Elche, Spain

**Keywords:** acne vulgaris, skin microbiota, gut microbiota, gut–skin axis, *Cutibacterium acnes*, probiotics, topical probiotics, skin inflammatory diseases

## Abstract

The objective of this narrative review was to check the influence of the human microbiota in the pathogenesis of acne and how the treatment with probiotics as adjuvant or alternative therapy affects the evolution of acne vulgaris. Acne is a chronic inflammatory skin disease involving the pilosebaceous units. The pathogenesis of acne is complex and multifactorial involving genetic, metabolic, and hormonal factors in which both skin and gut microbiota are implicated. Numerous studies have shown the bidirectionality between the intestinal microbiota and skin homeostasis, a communication mainly established by modifying the immune system. Increased data on the mechanisms of action regarding the relevance of *Cutibacterium acnes*, as well as the importance of the gut–skin axis, are becoming known. Diverse and varied in vitro studies have shown the potential beneficial effects of probiotics in this context. Clinical trials with both topical and oral probiotics are scarce, although they have shown positive results, especially with oral probiotics through the modulation of the intestinal microbiota, generating an anti-inflammatory response and restoring intestinal integrity, or through metabolic pathways involving insulin-like growth factor I (IGF-1). Given the aggressiveness of some standard acne treatments, probiotics should continue to be investigated as an alternative or adjuvant therapy.

## 1. Introduction

The epidermis, together with its adjoining structures such as the sweat and sebaceous glands, has an approximate surface area of 25 m^2^, which makes the skin one of the largest epithelial surfaces for interaction with microorganisms, along with the gastrointestinal tract. The skin is a first-line barrier against the external environment, and continuously interacts with it [1]. It is estimated that there are approximately 10^12^ bacteria on the skin, compared with 10^14^ of the intestinal microbiota [2,3].

During recent years, the immunomodulatory potential of the intestinal microbiota in remote organs has been increasingly investigated. Specifically, different degrees of influence of the gut microbiota on the gut–brain [4], gut–lung [5], gut–liver [6], and gut–skin axis [7,8] have been observed. Regarding the gut–skin axis, the gut microbiota basically modulates the functionality and composition of the innate and adaptive immune system, and vice versa [9]. This fact explains why some skin diseases have intestinal comorbidities [10], and suggests that there is a link between the presence of intestinal dysbiosis and the imbalance of skin homeostasis, with a special role of the intestinal microbiota in the pathogenesis of several inflammatory skin diseases [11]. Numerous studies have shown the bidirectionality between the intestinal microbiota and skin homeostasis, a communication established by modifying the immune system [12]. This modulation is mainly caused by the intestinal microbiota, but the cutaneous microbiota is also important in maintaining an adequate immune homeostasis of the skin, since it is also rich in immune cells (but not as many as the intestine, which houses 70% of the body’s immune cells) and is densely colonized by bacteria (but not as heavily as the large intestine) [13]. Thus, many studies have shown that the overgrowth of pathogens in the skin is a common finding in several skin diseases [7,8], although it is unknown whether this factor is a cause or consequence of such diseases.

Acne is a chronic inflammatory skin disease involving the pilosebaceous units that is characterized by the formation of comedones, papules, pustules, nodules, and scars that appear mainly on the face, upper trunk, and sometimes extremities.

The estimated prevalence of acne is difficult to estimate due to several factors. Different studies have not used the same definition of acne or the same scale of severity [14]. The estimated prevalence in the different studies was also strongly influenced by the country and sample size. For example, a study conducted in Turkey with 2300 individuals aged 13–18 years showed a prevalence of 60.7%. In this study, the diagnosis was established by dermatologists, and the degree of severity was established using the Pillsbury diagnostic criteria [15]. However, in another study conducted in Brazil with 2201 18-year-old males, the prevalence was estimated at 89.1%. In this second study, the diagnosis was made by dermatologists, but the degree of severity was established based on the number and type of inflammatory and noninflammatory acne lesions [16]. The Global Burden of Disease Study in 2010 estimated that acne, along with eczema, pruritus, and fungal skin disease, were the four most common skin disorders, with a global estimated prevalence of 8.96% in men and 9.81% in women (of any age) [17]. Onset of acne coincides with pubertal development and increased sebum production. Therefore, the prevalence is highest in adolescents and low in prepubertal children. After adolescence, the prevalence of acne decreases, although there is a percentage of adults who still have this disorder (up to 43% of subjects who had acne in adolescence still had acne at 30–40 years) [18]. It is significant that as one of the most prevalent skin disorders in developed countries, it hardly appears in hunter–gatherer communities without contact with the Westernized lifestyle [19]. This fact indicates the importance of lifestyle as a risk factor for the onset and development of acne. A strong correlation has been shown between age, family history of acne, body mass index (BMI), and skin type in the severity of acne presentation. Association with other factors such as smoking or diet are less consistent [14].

The pathogenesis of acne vulgaris is complex and multifactorial, and involves increased production of cutaneous sebum, hyperplasia of sebaceous glands under androgenic influence, ductal obstruction due to increased desquamation of keratinocytes, proliferation or presence of certain strains of *Cutibacterium acnes*, and infiltration of inflammatory cells [20]. It is difficult to establish the strength of each of these processes individually at the beginning and during development of this skin disorder.

The objective of this narrative review was to check the pathogenic mechanisms of acne, especially the influence of the human microbiota in the pathogenesis of acne, and how the modulation of this human microbiota through the use of probiotics as adjuvant or alternative therapy affects the evolution of acne vulgaris.

## 2. Pathogenesis of Acne Vulgaris

Acne is a follicular disease, and the initial comedone is caused by the impaction and distention of the follicles with a keratinous plug. A keratinous plug is caused by hyperproliferation and abnormal differentiation of keratinocytes. In addition, keratinocytes play a significant role in cutaneous immune function. They express various pattern recognition receptors (PRRs), including Toll-like receptors (TLRs) that can recognize a variety of bacteria. When keratinocytes are activated, they can produce antimicrobial peptides and cytokines, causing a direct antimicrobial effect as well as the recruitment and modulation of immune cells [21].

It is not fully established whether the hyperkeratinization of the follicle occurs before or after an inflammatory response is triggered [22]. Th17 lymphocytes present in acne lesions seem to play a key role in establishing an inflammatory process, resulting in the activation of derivate cytokines that cause neutrophilic infiltration and activation in the pilosebaceous follicle. They can also stimulate keratinocytes, endothelial cells, monocytes, and fibroblasts, producing proinflammatory mediators such as IL-6, TNFα, IL-1β, PGE2, nitric oxide, and matrix metalloproteinases [23]. Activation of the nuclear factor-kappa B (NF-κB) pathway is observed in acne lesions, along with secretion of cytokines derived [24]. Although in the following paragraphs we will develop the importance of the relationship between *Cutibacterium acnes* and the onset and development of acne, *C. acnes* can activate and induce expression of TLR2 and TLR4 [25]. Likewise, saturated fatty acids act as damage-associated molecular patterns (DAMPs), activating TLR2 and TLR4 and triggering the inflammatory response [26]. Therefore, the levels of certain potentially inflammatory lipids are abnormally increased in acne lesions. In fact, in sebocytes, the cyclooxygenase 2 (COX2) isoenzyme is selectively activated by inflammatory lipids in the sebaceous glands of acne patients, and is involved in the synthesis of prostaglandins [27]. For example, palmitic acid, a long-chain saturated fatty acid, can activate TLR2, producing heterodimerization with TLR1 [28]. Dimerization of TLR1 is a key process in the activation of the inflammasome in the pilosebaceous follicle in acne, which will cause the secretion of IL-1β. Along with this process, an increase in the expression of β-defensin-2 in sebocytes also is associated [29]. Likewise, palmitic acid is a powerful stimulator of the NLRP3 inflammasome of macrophages; therefore, the palmitic acid derived from sebum is a lipotoxic molecule that causes inflammation of the follicle [30]. The NLRP3 inflammasome is considered as a sensor that is activated by lysosomal rupture, as produced by palmitic acid and also *C. acnes* [31]. IL-1β is particularly important because it activates Th17 cells in acne lesions, which are responsible for the aforementioned effects.

Excess sebum is an essential contributor to the onset and development of acne and a risk factor for severe acne [14], although not all people with hyperseborrhea develop it. Sebum secreted by the sebaceous glands is an oily mixture that contains free fatty acids (FFAs) among other compounds such as squalene. Sebum can activate cell differentiation and proliferation, lipogenesis, and secretion of cytokines [32], and its production depends on several factors, including hormones [22]. Likewise, sebum of patients with acne presents alterations in the composition of FFAs [33], with a decrease in essential FFAs such as linoleic acid, as well as an association with proinflammatory lipids such as monounsaturated fatty acids (MUFAs) and lipoperoxides derived from the peroxidation of squalene of sebum. MUFAs and lipoperoxides contribute significantly to the hyperkeratinization of the pilosebaceous follicle [34].

Classically acne begins in adolescence, when there a considerable hormonal change occurs in both men and women. There is a systemic increase in androgen production of adrenal and gonadal origin and a local increase in androgen production at the skin level. Under normal conditions, the main circulating androgens are dehydroepiandrosterone sulfate (DHEA-S), dehydroepiandrosterone (DHEA), androstenedione, testosterone (T), and dihydrotestosterone (DHT). DHEA-S, DHEA, and androstenedione serve as prohormones of the effective hormones T and DHT, which interact with the androgen receptor present in many skin cells, such as sebocytes and follicular keratinocytes, generating an increase in sebum production and a proliferation of the sebaceous gland [35]. In the areas of the skin where acne lesions appear, there is a greater expression of the androgen receptor and the enzyme 5α reductase, which converts T into DHT, which is the most potent androgen [36,37]. Other signal molecules can activate other sebocyte receptors that lead to increased sebum production [38].

Insulin-like growth factor 1 (IGF-1) is a potent inducer of gonadal T and adrenal DHEA production, and promotes the intracutaneous conversion of T to DHT [20]. In addition, IGF-1 potently stimulates sebaceous lipogenesis and nuclear androgenic receptor signaling through the nuclear forkhead box-O1 (FoxO1) transcription factor [39]. A FoxO1 deficiency is widely evidenced as a key factor in the pathogenesis of acne, and a decreased expression of FoxO1 in this regard promotes lipogenesis, secretion of proinflammatory cytokines, and proliferation of keratinocytes [40]. Basically, the androgenic receptor of the sebaceous gland is activated in the presence of its ligands, being the one with the highest affinity the DHT, and by eliminating the repression exerted by the FoxO1 nuclear factor [41]. In addition, both insulin and IGF-1, through the activation of the phosphoinositol-3-kinase (PI3K)–protein kinase B (AKT) pathway, interact with the repressive function of FoxO1, allowing the activation of the androgenic receptors [23].

**Figure 1:**
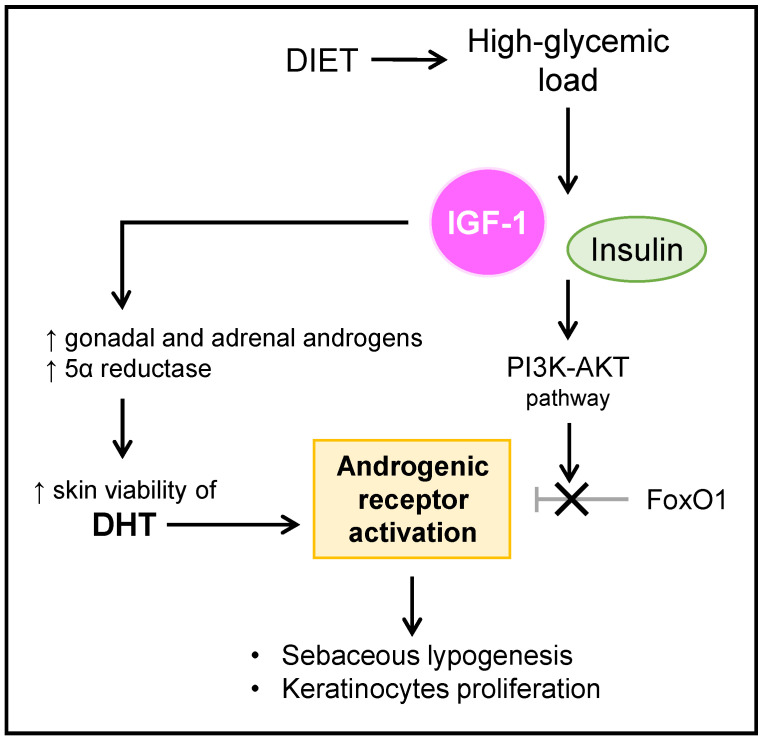
Influence of androgens and IGF-1 in acne. IGF-1: insulin-like growth factor 1; PI3K-AKT: phosphoinositol-3-kinase–protein kinase B (AKT); FoxO1: nuclear forkhead box-O1 transcription factor; DHT: dihydrotestosterone.

Figure 1 summarizes the mechanisms that involve the influence and relationship between androgens and IGF-1 that are established in acne patients.

Adult acne after adolescence can be persistent, recurring, or late-onset. It occurs more in women than in men [42]. In a study with more than 700 subjects over 25 years old, facial acne was observed in 12% of women and 3% of men [43]. In these women, there was an androgenic influence in which there may have been an increase in androgen production in the ovary or adrenal glands, an increase in androgen production in the skin, or an increase in the sensitivity of the androgen receptor [35]. It is probable that the formation of acne was more dependent on the local concentration of androgens or a greater sensitivity of sebocytes to them, and therefore the presence of acne in adult women would not correlate with serum levels of androgens. Sardana et al. observed in 120 women over 25 years old with late-onset (68 women) or persistent acne (52 women) that 72% had clinical hyperandrogenism, but only 18% had biochemical hyperandrogenism [44]. Therefore, in many adult women with symptoms of hyperandrogenism and acne, an increase in the concentrations of the main serum androgens were not observed. In women with menopause, acne is quite infrequent; however, when it occurs, it is usually in the form of comedones [45]. With the entry into menopause, there is a drastic reduction in estrogens; however, androgens experience a gradual decrease. At this age, acne appears mainly in the context of postmenopausal hyperandrogenic disorders [46]. 

Acne in adult men is much less common than in women, and the underlying mechanisms have not been studied. Increased androgen-receptor sensitivity or excessive production of DHT is likely to occur.

It is a fact that excessive androgens intervene in an important way in the development of acne. Even so, acne does not develop when there is loss of functionality of the androgen receptor [47], but IGF-1 is the main hormone that controls pubertal development, and decreases after this phase [48]. These issues focus on IGF-1 as a key molecule in the onset and development of acne. An enlightening fact is that patients with Laron syndrome who have growth hormone receptor (GHR) mutations have an IGF-1 deficiency, and if they are not treated with IGF-1 recombinant, they never develop acne. However, when they are treated, they develop acne [49]. However, for the establishment of acne, androgen-mediated sebum production is necessary but not sufficient. This was confirmed by studies that evaluated the efficacy of 5α-reductase inhibitors that did not significantly improve acne [50,51].

**Figure 2:**
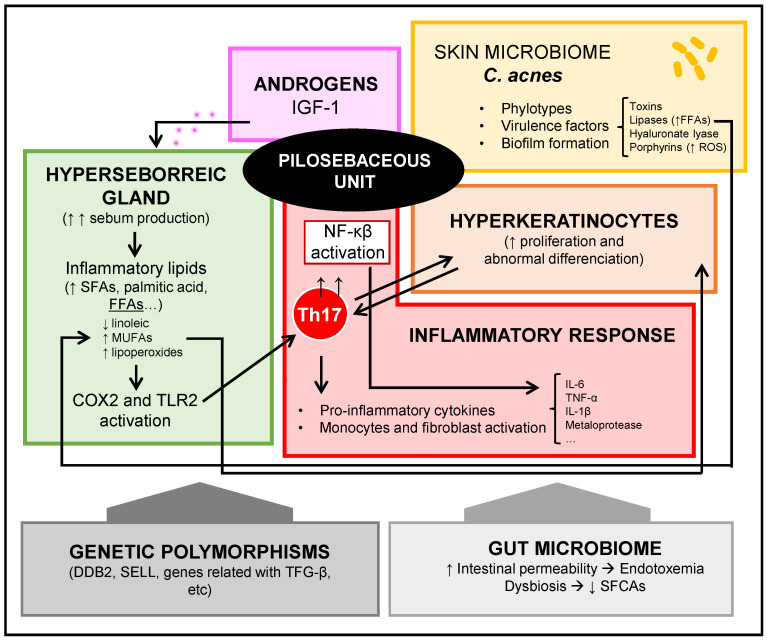
Pathogenic mechanisms of acne. IGF-1: insulin-like growth factor 1, FFAs: free fatty acids, ROS: reactive oxygen species, SFAs: saturated fatty acids, MUFAs: monounsaturated fatty acids, COX2: cyclooxygenase 2, TLR2: Toll-like receptor 2, Th17: Th17 cells, NF-κB: nuclear factor-kappa B, TGF-β: transforming growth factor receptor β, SCFAs: short-chain fatty acids.

An association with genetics in the presentation of acne has been demonstrated, since family history is a clear significant risk factor [14]. In extensive case-control studies conducted in different regions and with ethnic differences, several associations of genetic polymorphisms and presence of acne have been evidenced. The conclusions that can be drawn from these studies include that there is a variability in genes associated with acne between different regions and ethnicities. In 2014, He et al. observed 1056 cases with severe acne and controls from a Chinese population, and found an association between acne and DDB2 (DNA-binding protein 2) and SELL (L-selectin) genes [52]. These genes participate in androgen metabolism and inflammation, respectively. Navarini et al. compared 1893 severe cases of acne and 5132 controls from the United Kingdom in 2014, and found that there was an association between acne and genes related to the transforming growth factor receptor (TGF)-β signaling pathway, such as OVOL1, FST, and TGFβ2 [53]. The TGF-β signaling pathway participates in tissue remodeling and appearance of scars. However, in 2017, Mina-Vargas et al. observed 4491 twins and their siblings in Australia, and did not find a statistical association between genes and acne severity, although three genes were associated with the presence of acne [54]. These genes, PTPRU, PIK3R1, and DCC, participate in cell growth, differentiation, and adhesion, respectively. In general, genes associated with acne participate in the regulation of the immune response or androgen metabolism [55]. In 2022, Mitchell et al. published a meta-analysis of a genome-wide association study (GWAS) with 20,165 subjects with acne from nine historical European cohorts, and identified 29 new related loci. Many of these acne-related loci influence hair follicle structure and morphology, development and maintenance of the pilosebaceous unit, and neutrophil infiltration of lesions [56]. Figure 2 summarizes the different pathogenic mechanisms that are established in the onset and development of acne.

## 3. Acne Vulgaris and Skin Microbiota: *Cutibacterium acnes*

Studies on the skin microbiota in acne pathogenesis have focused on the influence of *C. acnes* on the onset and development of acne. The first study that reported the presence of this bacteria in acne lesions was that of T.C. Gilchrist in 1900 [57]. However, the role of *C. acnes* is unclear, as it is a ubiquitous bacteria in healthy human skin [2]. Therefore, while *C. acnes* is a predominant bacteria in sebaceous regions of the skin, with a vital role in cutaneous homeostasis, even acting in the prevention of pathogens [58], it can also act as an opportunistic pathogen. In recent years, there have been many studies that have contributed to gradually clarifying the pathogenic mechanisms of *C. acnes* in acne patients.

First, it is important to clarify the distinct categories of *C. acnes* phylotypes based on different methodologies, because this point can be somewhat confusing. Initially, by genomic comparison of the hemolysin gene (recA or tly), the phylotypes IA1, IA2, IB, II, and III were established [59]. Then, researchers used multilocus sequence typing (MLST) to obtain the phylotypes IA1, IA2, IB, IC, II, and III, and within these they located different clonal complexes (CC) [60]. These classifications are important, since recent studies suggest that *C. acnes* acts as a potential commensal or pathogen, depending on the type of strain and the presence of some metagenomic elements [61]. It is remarkable to note that *C. acnes* is the most abundant bacteria in the pilosebaceous follicle both in healthy patients and in patients with acne, and that there are no differences in terms of bacterial load, so it was proposed to investigate the functionality of *C. acnes* instead of just its presence in acne lesions [62]. Several studies have focused on this approach that resulted in several consistent findings. In 2010, Lomhol et al. conducted the first study to demonstrate that distinct phylotypes of *C. acnes*, specifically subgroup IA, were significantly associated with moderate to severe acne [63]. In 2013, Kwon et al. observed that although the distribution of phylotypes was similar between healthy skin and comedones, there was an increase in phylotype IA in papules and pustules [64]. Using MLST, it was observed that there were 1 to 10 strains coinciding, and that in acne lesions, all strains came from phylotype IA1, and almost exclusively from CC18 [65]. McDowell’s group in 2013 devised a more refined version of their classification of *C. acnes* subgroups using MLST based on only four genomic markers [66]. They demonstrated that CC1, CC2, and CC3 of phylotype IA1 dominated acne lesions, while the rest of the subgroups were observed much less frequently or even not observed. However, other studies did not identify a significant relationship between different phylotypes and severity of acne [67]. Therefore, it is more likely that the severity of acne is due to a greater inflammatory response, and this depends in part on *C. acnes*, but also on other crucial factors [68] such as an excess of sebum with alteration in its lipid composition, hormonal influence, immune response influenced by the intestinal microbiota, and genetic predisposition, among others.

Another critical issue is the presence of *C. acnes* with virulence factors in acne lesions [61]. These virulence factors could explain the different frequency of appearance of different phylotypes in acne lesions. In 2013, Tomida et al. sequenced the complete genome of 82 strains of *C. acnes* and observed that it had a constant core region and a variable region. This variability would explain its commensal or pathogenic phenotype and its influence on the development of acne [69].

Let us review the important virulence factors of *C. acnes*.

There are five Christie–Atkins–Munch–Petersen (CAMP) factors identified in the genome of all *C. acnes*. These are secreted toxins that cause the formation of pores in the membranes and are potentially cytotoxic to keratinocytes and macrophages, causing inflammation of the skin [70]. CAMP1 was associated with phylotypes IB and II, while CAMP2 was associated with IA [71]; however, it was recently shown that CAMP1 was also present in phylotype IA1 [72]. CAMP1 interacts with TLR2, but the potency of inflammatory response depends on the phylotype. In this way, phylotype IA causes a low inflammatory response, while it is more powerful when caused by the phylotypes IB and II. This indicates that CAMP1 is not the only virulence factor that causes inflammation, because the phylotype IA is more associated with acne. It is important to emphasize that CAMP1 presents a high genetic variability, although it is unknown how this affects virulence, and there could be a relationship [73].

In 2016, Johnson et al. showed that *C. acnes* phylotypes more related to acne produced more porphyrins, which are metabolites that generate reactive oxygen species (ROS) and can lead to inflammation in keratinocytes [74]. In this regard, phylotype IA1 strains produced higher amounts of porphyrins than phylotype II strains. The fact that ROS was generated would activate inflammatory processes such as lipid peroxidation of sebum lipids such as squalene [34]. Vitamin B12 supplementation could be counterproductive in acne patients, since virulent strains of *C. acnes* associated with acne produce porphyrins from this vitamin [75].

Hyaluronate lyase is an enzyme capable of degrading hyaluronic acid and other glycosaminoglycans from the extracellular matrix of skin cells, contributing to inflammation in acne lesions. There are two variants of hyaluronate lyase found in *C. acnes*: HYL-IB/II and HYL-IA. HYL-IB/II causes a complete degradation of hyaluronic acid by being more active, while HYL-IA performs an incomplete degradation. HYL-IB/II is present in phylotypes IB and II, and HYL-IA in phylotype IA. Whereas type IA strains are primarily found on the skin surface and are associated with acne vulgaris, type IB/II strains are more often associated with soft and deep tissue infections, which would require elaborate tissue-invasion strategies, possibly accomplished by a highly active HYL-IB/II [76]. 

Lipases are enzymes that metabolize lipids of the sebum, generating FFAs. It has been shown that *C. acnes* encodes 12 lipases, including glycerol-ester hydrolase A (GehA) and B (GehB), which are present in strains of *C. acnes* present in healthy skin and acne lesions [77]. *C. acnes* lipases are important in the context of acne because they are important to the growth of this bacteria in lipophilic environments (pilosebaceous unit) [78], and because by producing FFAs, they promote inflammation [33]. However, the exact role of the different *C. acne* lipases in the onset and development of acne is partly unknown.

Other potential virulence factors of *C. acnes* described and associated to a greater or lesser extent with acne are the enzymes neuraminidase, PUFA isomerase, gliycosidase, sortase F, and RoxP [70,79]. Likewise, heat-shock proteins [80] and dermatan-sulfate-binding adhesins have also been studied [70].

An important fact regarding strains that belong to phylotype II of *C. acnes*, which is most associated with healthy skin, is the presence of CRISPR/Cas sequences, which allows these bacteria to eliminate several genetic materials acquired horizontally. Nevertheless, phylotype I strains most associated with acne lesions usually present deletions in regions of CRISPR/Cas sequences, which implies a greater capacity to acquire virulence factors through horizontal transmission [81].

A particularly important virulence factor of *C. acnes* related to therapeutic failure of antibiotics in patients with acne is the ability to form biofilms. A bacterial biofilm is a structured colony of bacterial cells embedded in a polymer matrix that is manufactured by the bacteria and adhered to a surface. These biofilms can be located in the pilosebaceous follicle and favor the development of comedones, increase adhesion of *C. acnes* to the walls of the follicle, and increase the compacting of keratinocytes [82]. Formation of biofilms by *C. acnes* is due to the presence of genes involved in such formation, such as glycosyltransferases and uridine diphosphate N-acetylglucosamine 2-epimerase [83]. The relative frequency of appearance of biofilms formed by *C. acnes* is significantly higher in acne lesions than in the skin of healthy controls [84]. It is important to note that the biofilm of *C. acnes* presents factors that cause or aggravate inflammation such as CAMP, as has been shown in in vitro models [85]. The presence of biofilms is associated with a greater resistance to antibiotics because this exopolysaccharide matrix prevents or hinders the interaction of antibiotic to its molecular target. Acne treatment is partly based on antibiotic therapy, and many therapeutic failures can be attributed to the presence of *C. acnes* biofilms in acne lesions. In vitro studies have shown that markedly high concentrations of antibiotics are needed to inhibit growth or eradicate *C. acnes* in its biofilm [86]. However, some antibiotics, such as penicillin, penetrate the *C. acnes* biofilm better than others, such as ciprofloxacin or clindamycin [87].

In general, strains of *C. acnes* more associated with acne induce a more potent inflammatory response than less-associated strains [88]. However, any cell that is part of pilosebaceous follicle, such as keratinocytes, sebocytes, or macrophages, interacts with *C. acnes*, and this interaction is mainly through TLRs [89]. The TLRs contain an intracellular domain that allows production of cytokines and other proinflammatory molecules, as well as an extracellular domain involved in the recognition of pathogen-associated molecular patterns (PAMPs) and DAMPs. *C. acnes* activates the innate immune system by interacting with TLR2 and TLR4, so that NF-κB and MAPK pathways and NLRP3 inflammasome are activated. Consequently, *C. acnes* induces production of proinflammatory cytokines such as IL-1α, IL-1β, IL-6, IL-8, IL-12, and TNFα, as well as other proinflammatory molecules such as matrix metalloproteases, as evidenced by in vitro studies [90]. Matrix metalloproteases contribute to tissue-remodeling processes after inflammation, resulting in the appearance of scars typical of moderate and severe acne. In addition, as we showed in the previous section on acne pathogenesis, *C. acnes* also acts at the level of adaptive immunity. An infiltration of CD4 cells has been evidenced in the pilosebaceous follicle in the initial stages of acne; CD3, CD4, and macrophage cells were found from the beginning of formation of the microcomedone [91]. These data would indicate that the inflammatory process could appear before hyperkeratinization. In addition, as we mentioned previously, activation of Th17 cells by different mechanisms is a key process, because these T cells present in acne lesions play a key role in establishing an inflammatory process, resulting in the activation of derivate cytokines that cause neutrophilic infiltration and activation in the pilosebaceous follicle [23].

## 4. Acne Vulgaris and Gut Microbiota

The exact mechanisms and their contribution to the influence of the gut microbiota on the onset and development of acne are not established. By analogy with immune-based inflammatory diseases such as atopic dermatitis or psoriasis [7,8] and with evidence of the existence of the gut–skin axis, it is likely that the main mechanism participates in the inflammatory immune response [13].

A possible influence of the gut microbiota in acne may be an interaction with the mTOR (mammalian target of rapamycin) pathway. Recent studies have shown an impaired mTOR pathway in various skin diseases [92]. In general, mTOR is a nutrient-sensitive regulator that participates in processes of cell growth and differentiation in the skin, being key in homeostasis and in forming an appropriate epidermal barrier [93]. Mistakes in the mTOR pathway negatively affect these processes in different skin diseases. In 2016, Monfrecola et al. evidenced for the first time the role of the mTOR pathway in the pathogenesis of acne, as they observed an increase in the expression of mTOR in patients with this skin disease, both in affected skin and in unaffected skin, compared to healthy controls [94]. In 2016, Agamia et al. found increased serum IGF-1 concentrations, higher cytoplasmic expression of FoxO1, and more intense cytoplasmic and nuclear expression of mTOR in acne patients than in healthy controls, and an excessive consumption of a high-glycemic-load diet was associated with higher serum levels of IGF-1 and cytoplasmic expression of FoxO1 and mTOR [95]. Likewise, studies in mice have shown that by causing glucose intolerance and obesity through a diet with a high glycemic load that modified mTOR activity, it was related to specific changes in composition of gut microbiota that were evidenced inversely by administering resveratrol (specific inhibitor of mTOR complex 1) to these mice [96]. These studies linked to the effects of diet, intestinal microbiota, and pathogenesis of acne related to the mTOR pathway. In addition, bacterial metabolites can interact with the mTOR pathway, and the mTOR pathway can also affect the gut microbiota by controlling the intestinal barrier [97]. Therefore, if an altered intestinal barrier is established in the context of intestinal dysbiosis, a positive feedback loop can form, which can amplify inflammation and aggravate acne.

Studies from many years ago already showed that endotoxemia and increased intestinal permeability can be increased in acne. In 1916, Strickler et al. suggested that intestinal permeability may be increased in patients with acne because a complement fixation test demonstrated increased serum reactivity to fecal coliforms in 66% of patients versus 0% of controls [98]. In 1930, Stokes et al. identified an association between the gut microbiota and skin inflammation. In their study, up to 40% of acne patients had hypochlorhydria, and they hypothesized that a lack of gastric HCl could lead to migration of bacteria from the colon to the small intestine [99]. Today we know that small intestinal bacterial overgrowth (SIBO) can cause an increase in intestinal permeability (leaky gut), leading to systemic inflammation [100]. Likewise, in 1983 Juhlin et al. observed patients with acne of different degrees of severity and detected lipopolysaccharide (LPS) from *Escherichia coli* in serum in 5 of 10 subjects with severe acne, but not in subjects with moderate acne or in controls [101]. When the intestinal barrier is altered, gut microbiota and its metabolites reach the circulation, accumulate in the skin, and can alter skin homeostasis [13].

There have only been a few studies that investigated the gut microbiota in acne patients. The first attempt to assess the gut microbiota in acne patients was a study by Loveman et al. in 1955 [102]. Stool cultures were grown from 10 patients with severe pustular acne vulgaris, and no significant differences were found in a small classic group of intestinal pathogenic bacteria compared to a group of 10 healthy controls. 

The first larger study that found differences in the intestinal microbiota of acne patients using microbiological culture techniques was that of Volkova et al. in 2001 [103]. In 114 patients with acne (94 with papulopustular acne and 20 with nodulocystic acne), 54% presented gut dysbiosis.

The following studies in this regard are recent and were already based on the next-generation sequencing (NGS) of the bacterial 16S rRNA gene. In 2018, Deng et al. studied the intestinal microbiota of 43 treatment-naïve patients with different degrees of acne versus those of 43 age- and sex-matched healthy controls. A decreased alpha diversity was observed in acne patients compared to healthy controls, with no differences in severity. A principal coordinate analysis (PCoA) showed two different clusters for cases and controls, which was confirmed by an analysis of similarities (ANOSIM) test of the UniFrac distances between both groups. A linear discriminant analysis effect size (LefSe) analysis found 38 differences between the groups of acne patients and healthy controls. At the phylum level, there was a decrease in Firmicutes and an increase in Bacteroidetes in patients with acne. There was a remarkable decrease in Clostridial families such as Lachnospiraceae and Ruminococcaceae, which are potentially beneficial groups and producers of short-chain fatty acids (SCFAs), in the acne patients. In addition, a PICRUSt analysis was performed that showed an increase in LPS synthesis pathways and a decrease in the metabolism of glycerolipids and glycerophospholipids in patients with acne [104].

More recently, Deng’s researcher group conducted a study in which they analyzed possible discrepancies in the intestinal microbiota and fecal metabolites between men and women with acne, since it is unknown whether gut dysbiosis and its associated metabolism are sex-dependent in subjects with acne [105]. Alpha diversity was lower in men with acne compared to control men, but no significant differences were found between women with acne and control women. An ANOSIM test showed four statistically significant clusters among the four study groups: men with acne, control men, women with acne, and control women. At the phylum level, Firmicutes and Bacteroidetes were significantly increased and decreased, respectively, in men with acne versus healthy control men. These differences were not found in women with acne versus healthy control women. At the genus level, decreases were observed in men with acne compared to healthy control men, including 18 genera such as *Lysinibacillus*, *Paenibacillus*, *Aerococcus*, *Alkaliphilus*, *Carnobacterium*, *Lactococcus*, *Oceanobacillus*, *Bacillus*, *Blautia*, *Butyricicoccus*, *Gemmiger*, *Lachnospiracea_incertae_sedis*, *Exiguobacterium*, *Pseudomonas*, *Enterococcus*, *Faecalibacterium*, *Bilophila*, and *Ruminococcus*. In women with acne compared to healthy control women, an increase in *Clostridium sensu stricto* and a decrease in *Oscillibacter* and *Odoribacter* were observed. Likewise, men and women had different changes in fecal metabolites with respect to controls. Men with acne tended to have an impaired fatty acid metabolism, while women with acne tended to have an impaired amino acid metabolism. Specifically, an increase in long-chain fatty acids such as alpha-linolenic acid (omega-6) was observed. Omega-6 are long-chain fatty acids with proinflammatory activity, and aggravate the severity of acne [106]. This study showed for the first time that men and women with acne had different types of gut dysbiosis and different associated metabolites. In this way, men who presented more severe cases of acne presented an intestinal microbiota with less alpha diversity and richness, with losses of beneficial anti-inflammatory SCFA-producer bacteria such as *Faecalibacterim*, and others such as Clostridiales [107].

In 2018, Yan et al., in a case-control study like the one conducted by Deng [104], showed fewer differences in the gut microbiota of acne patients compared to healthy controls [108]. No differences were observed regarding alpha diversity between cases with acne and healthy controls, and a principal component analysis (PCA) did not show different clusters. Therefore, the intestinal microbiota between cases and controls was quite similar, although a loss in *Bifidobacterium* and a slight gain in Proteobacteria were observed in patients with acne (statistically significant differences). *Bifidobacterium* can balance gut microbiota by fermenting indigestible oligosaccharides and inhibiting the growth of pathogenic bacteria, which improves the intestinal barrier, decreases the intestinal permeability, and induces Treg (CD4+Foxp3+) cell generation [109]. Changes in *Bifidobacterium* and Proteobacteria (increased LPS, increased intestinal permeability) may aggravate or cause the development of acne in these patients.

In 2020, Thompson et al. conducted a small case-control study with eight patients with acne receiving minocycline as treatment and eight healthy age- and sex-marched controls without treatment [110]. No significant differences in alpha diversity were found between acne patients before and after antibiotic therapy. The intestinal microbiota of patients with acne before taking antibiotics compared to those of healthy controls showed a decrease in the species *Lactobacillus iners*, *Lactobacillus zeae*, and *Bifidobacterium animalis*. After antibiotic treatment, the gut microbiota of acne patients showed decreases in *Lactobacillus salivarius*, *Bifidobacterium adolescentis*, *Bifidobacterium pseudolongum*, *Bifidobacterium breve*, and *Akkermansia mucinophila* compared to healthy controls. An increase in Bacteroidetes (even before treatment, Bacteroidetes was already higher than in healthy controls) was observed in acne patients after treatment (causing a decrease in the Firmicutes/Bacteroidetes ratio). Therefore, minocycline caused changes in the intestinal microbiota (at the level of potential probiotic species from *Lactobacillus* and *Bifidobacteirum* genera). This small study highlighted the potential relevance of adjuvant probiotic therapies. In line with these results, in another small study with eight subjects with acne after 4 weeks treatment with minocycline and eight age-, race-, and sex-matched healthy controls, it was observed that the Firmicutes/Bacteroidetes ratio was higher in controls and lower in patients with acne after treatment. In patients with less-severe acne, a slightly higher baseline Firmicutes/Bacteroidetes ratio was observed compared to those with greater severity [111].

The studies that have evaluated or provided information on the relationship between acne and the intestinal microbiota are summarized in Table 1.

## 5. Acne Vulgaris and Probiotics

Human clinical trials that used probiotics for the treatment of acne have been scarce. However, in vitro studies have shown several interesting properties of some probiotic strains regarding the pathogenesis of acne. Most in vitro studies have focused on evaluating the ability of probiotic strains to produce antimicrobial substances that inhibit the growth of *C. acnes* through various mechanisms. 

*Streptococcus salivarius* [112], *Lactococcus* sp. HY 449 [113], and *Lactobacillus salivarius* LS03 [114] produce bacteriocins that inhibit the growth of *C. acnes*. *Bifidobacterium adolescentis* SPM0308 was effective in controlling growth of both *C. acnes* and *S. aureus* due to its antimicrobial activity [109]. 

Wang et al. showed the effects of *Staphylococcus epidermidis* in inhibiting the growth of *C. acnes* due to its effects on glycerol fermentation. It was shown that a product of glycerol fermentation, succinic acid, was responsible for the inhibitory effects on *C. acnes*, which was also verified in a murine model [115]. 

Cosseau et al. showed that *Streptoccocus salivarius* K12 stimulated an anti-inflammatory response and modulated genes associated with epithelial adhesion, which could help it to remain on the epithelial surface while protecting it from pathogen-induced inflammation and apoptosis [116]. 

Likewise, Gueniche et al. showed that *Lactobacillus paracasei* CNCM I-2116 had beneficial effects on key mechanisms associated with the skin barrier function, such as the release of TNF-α induced by substance P [117]. 

Another mechanism that in vitro studies have shown to be relevant in terms of the inhibition of the growth of *C. acnes* is that of the formation of ceramides. Ceramides can retain water in the skin, and some of them, such as phytosphingosine, have even shown antimicrobial properties against *C. acnes* [118]. Some probiotics, such as *Streptococcus thermophilus*, increase the production of ceramides [119]. 

In addition, the growth-inhibitory capacity of *C. acnes* of probiotic strains *Lactobacillus casei* NCFB 161, *Lactobacillus acidophilus* NCFB 1748, *Lactobacillus plantarum* DSM 12028, *Lactobacillus gasseri* NCFB 2233, and *Lactococcus lactis* NCIMB 66 together with glucomannan hydrolysates of *Amorphophallus konjac* has been shown [120]. 

Interestingly, in 2017, Lopes et al. observed that many strains of *Lactobacillus* and *Bifidobacterium* had a good ability to adhere to keratin and to inhibit biofilm formation of pathogenic bacteria, but a limited ability to adhere to *C. acnes* [121], and this fact could be relevant to their potential use as topical probiotics.

Recently, in 2021, two in vitro studies showed the possible benefits of strains of *Lactiplantibacillus plantarum* and *Weisella viridescens* in the pathogenesis of acne. Chae et al. evidenced antimicrobial effects produced by *Lactiplantibacillus plantarum* APsulloc 331261 and APsulloc 331266 at the skin-pathogen level [122]. Espinoza-Monje et al. conducted in vitro studies in which they observed that *Weissella viridescens* UCO-SMC3 inhibited the growth of clinical isolates of *C. acnes* and reduced the adhesion of this pathogen to keratinocytes. Furthermore, in a murine model, *Weissella viridescens* UCO-SMC3, both administered orally and topically, was shown to beneficially modulate the immune response against *C. acnes* and reduce *C. acnes* replication in lesions. Even orally, *Weissella viridescens* UCO-SMC3 produced a more potent anti-inflammatory response than topically [123].

The first clinical trial with probiotics in acne patients was conducted by R. Siver in 1961 [124]. A mixture of *Lactobacillus acidophilus* and *Lactobacillus bulgaricus* was administered orally to 300 acne patients for 8 days, followed by 2 weeks of washout and then 2 more weeks of treatment. Different degrees of clinical improvement were observed in 80% of acne patients, and this intervention was more effective in cases of inflammatory acne.

In 2010, Kim et al. evaluated the effects of a lactoferrin-enriched fermented milk compared to fermented milk only in 36 acne patients over 12 weeks [125]. A clinical improvement was observed in patients with acne who were administered lactoferrin-enriched fermented milk. The number of inflammatory lesions, number of total lesions, degree of acne severity, sebum content, and amount on skin surface of triacylglycerols and free fatty acids decreased significantly after treatment. The authors highlighted supplementation with lactoferrin, a protein that has been shown to have anti-inflammatory and bactericidal properties in vitro [126]. 

In 2013, Jung et al. conducted a clinical trial on 45 acne patients who were divided into three groups [127]. One group was treated orally with a probiotic mixture, another with the antibiotic minocycline orally, and the third one with the probiotic mixture plus minocycline orally. The duration of treatment in all cases was 12 weeks. The probiotic mixture contained different strains of *Lactobacillus acidophilus*, *Lactobacillus bulgaricus*, and *Bifidobacterium bifidum*. All three groups of patients showed improvement in the number of total lesions at 4 weeks, and continued to show improvement until the end of the study. Starting at week 8, patients taking a probiotic mixture plus minocycline had significantly better efficacy in terms of total number of lesions than the other two groups. The authors concluded that adjuvant treatment with a probiotic mixture elicited synergistic anti-inflammatory effects and reduced potential adverse effects of prolonged antibiotic therapy. 

A double-blind, placebo-controlled, randomized clinical trial in 20 adult subjects to assess the efficacy of the oral probiotic *Lactobacillus rhamnosus* SP1 was conducted by Fabbrocini et al. in 2016 [128]. After 12 weeks of treatment, changes in expression of IGF-1 and FoxO1 genes were compared in skin areas with acne lesions. In the probiotic group, there was a statistically significant reduction in the expression of the IGF-1 gene of 32%, as well as a statistically significant increase in the FoxO1 gene of 65%. No significant changes were seen in the placebo group. In addition, there was a considerable clinical improvement in the patients who were treated with the probiotic. The authors speculated that exact mechanisms by which the probiotic produced beneficial effects by normalizing cutaneous expression of IGF-1 and FoxO1 genes were unknown, although it is possible that this probiotic strain improved insulin resistance through a metabolic effect directly and/or by restoring an established intestinal dysbiosis in patients with acne. 

Recently, Rahmayani et al. investigated the effects of a probiotic mixture administered orally on IL-10 levels in 33 acne patients after 30 days of treatment [129]. The probiotic mixture contained the strains *Bifidobacterium lactis* W51, *Bifidobacterium lactis* W52, *Lactobacillus acidophilus* W55, *Lactobacillus casei* W56, *Lactobacillus salivarius* W57, and *Lactococcus lactis* W58. An increase in levels of the anti-inflammatory IL-10 [130] was observed after treatment with the probiotic mixture. 

Another double-blind clinical trial was conducted in men with mild to moderate acne who were treated with an oral supplement containing probiotics, biotin, vitamin E, zinc, nicotinamide, β-sitosterol, and *Boswellia serrata* extract [131]. After 12 weeks of treatment, these patients presented clinical improvement based on the reduction of the Global Acne Grading System (GAGS) score. The *Escherichia coli* Nissle 1917 strain has also been used in clinical trials in acne patients. 

In 2016, Manzhalii et al. administered this strain in 82 patients with intestinal-borne dermatoses (some of them were diagnosed with acne, and the rest with papular-pustular rosacea and seborrheic dermatitis) [132]. One group of patients was treated with a conventional topical therapy, and other with the probiotic strain administered orally for one month. A total of 89% of the patients treated with *E. coli* Nissle 1917 improved significantly, while 56% improved in the group treated with the conventional therapy. By studying the composition of gut microbiota and other parameters, the authors concluded that the *E. coli* Nissle 1917 strain, by protecting intestinal permeability and restoring intestinal microbiota, produced these beneficial effects. 

Finally, in 2002, Rinaldi et al. evaluated the efficacy of a mixture of the probiotic strains *Bifidobacterium breve* BR03 DSM 16604, *Lacticaseibacillus casei* LC03 DSM 27537, and *Ligilactobacillus salivarius* LS03 DSM 22776 plus a botanical extract of *Solanum melongena* and *Echinacea* administered orally in 114 patients with mild to moderate acne for 8 weeks through a randomized, placebo-controlled clinical trial [133]. A decrease in the number of acne lesions, rate of desquamation, rate of sebum secretion, and presence of *C. acnes* was observed in patients who were treated with the probiotic mixture and the botanical extract, as well as the mixture of both, with respect to placebo treatment. The stronger effects were seen with the probiotic mix plus the botanical extract.

Not only orally administered probiotics, but also topical probiotics for skin diseases, and especially for acne, have been considered. Topical probiotic treatment is considered to be safe and without adverse effects, especially when compared to standard therapy, which can sometimes be more aggressive [134]. Despite the increase in the commercial supply of products with supposed beneficial effects for acne based on probiotics in a topical pharmaceutical formula, the truth is that there have not been many clinical trials in this regard, and the current evidence on its effectiveness is scarce. Mechanisms of action of topically administered probiotics are partly unknown, but are intuited mainly due to preclinical in vitro studies [109,112,113,114,115,116,125]. The main mechanisms of action of topical probiotics described by the authors of these studies were improving the barrier function of the skin and secreting antimicrobial substances that inhibited the growth of *C. acnes*.

In 1912, J. Peyri conducted an experiment regarding the application of topical formulas based on probiotics such as *Lactobacillus bulgaricus* in different skin diseases, including acne, and highlighted their potential therapeutic effect in the inhibition of skin pathogens [135]. 

In 2009, Kang et al. conducted a double-blind, randomized, placebo-controlled clinical trial with an 8-week duration in 70 patients with acne using a concentrated powder lotion obtained from the supernatant of a culture of *Enterococcus faecalis* SL-5, a bacteria that produces the bacteriocin ESL5 [136]. Patients who were treated with this lotion significantly decreased inflammatory lesions compared to a placebo lotion. 

In another clinical trial from 2017, the ammonia-oxidizing bacteria *Nitrosomonas eutropha*, was used in 358 adult patients with mild or moderate acne, and a significant reduction in overall severity was observed after 12 weeks of treatment, as well as a trend in the reduction in the number of inflammatory lesions compared to the control group [137]. Ammonia-oxidizing bacteria convert ammonia to nitrite, which has antibacterial properties on the skin, and to nitric oxide, which regulates inflammatory and vasodilation processes. 

In 2022, Sathikulpakdee et al. conducted a randomized clinical trial to evaluate the efficacy of a probiotic-derived lotion versus a 2.5% benzoyl peroxide lotion in 104 patients with mild to moderate acne after 4 weeks of treatment [138]. The probiotic-derived lotion was obtained from supernatant of a culture of *Lactobacillus paracasei* MSMC 39-1, a bacteria that has been shown to inhibit the growth of *C. acnes*. In both groups, acne lesions and erythema index decreased; therefore, the lotion derived from *L. paracasei* MSMC 39-1 would be a safe alternative comparable to the 2.5% benzoyl peroxide lotion.

Currently, there are two clinical trials of acne patients receiving oral probiotics in the recruitment process registered at https://clinicaltrials.gov (accessed on 3 May 2022): two randomized, double-blind, placebo-controlled clinical trials in 80 and 70 patients, respectively, with codes NCT04570319 and NCT04596748.

In vitro studies, animal models, and clinical trials with oral and topical probiotics are summarized in Table 2.

## 6. Acne Vulgaris and Diet

Intimately related to the composition and diversity of intestinal microbiota, diet is a decisive factor in the development of acne. A Western diet characterized by a high consumption of ultraprocessed foods, saturated fats, and refined sugars is a risk factor for acne aggravation. A recent systematic review has shown that foods with a high glycemic index/load, dairy products, fatty foods, and chocolate promote the formation of acne lesions, while the intake of fruits and vegetables was protective [139].

A high-fat diet (HFD) implies a loss of diversity of gut microbiota and an increase in endotoxemia, which contributes to the development of a deterioration in the integrity of the intestinal epithelium and its barrier function, a decrease in the thickness of the intestinal mucosa layer, and an increased secretion of proinflammatory cytokines. Inflammation and the innate immune system are key factors in the pathogenesis of acne [79]. 

Observational studies have positively correlated a high glycemic index with acne [140]. Many foods in the Western diet with a high glycemic load increase IGF-1 and insulin levels, which affects the expression of FoxO1, along with the consequences that this entails, as we reviewed in previous sections. Basically, a FoxO1 deficiency is a key factor in the pathogenesis of acne and promotes lipogenesis, secretion of proinflammatory cytokines, and proliferation of keratinocytes [23]. Thus, it is important to highlight the importance of the sterol regulatory element-binding protein (SREBP)-1, which is critically regulated by FoxO1 [141]. SREBP-1 regulates the expression of stearoyl-CoA desaturase and ∆6-desaturase. Stearoyl-CoA desaturase catalyzes the conversion of stearic acid to oleic acid, the main fatty acid of sebum triglycerides, and ∆6-desaturase is key to the synthesis of unsaturated fatty acids, precursors of proinflammatory eicosanoids [23]. 

A Western diet is also extraordinarily rich in red meat, meanwhile vegetarian and vegan diets include reduced or no intake of meat and dairy. Meat/dairy-protein-based diets provide more leucine than vegetarian or vegan diets. Leucine stimulates the mTOR pathway, which supposes an increase in lipogenesis of the sebaceous gland [142]. Because leucine activates mTORC1, those who consume meat/dairy-protein-based diets increase that activation, possibly aggravating the inflammation implicated in acne [143]. Likewise, diets rich in animal protein decrease the diversity of the intestinal microbiota, while plant-based diets increase it [144]. This could also influence the onset and development of acne because the gut microbiota influences the shaping of the inflammatory response [145].

## 7. Conclusions

Acne vulgaris is a mechanistically complex multifactorial disease involving genetic, metabolic, and hormonal factors in which both the skin and gut microbiota are implicated. Increased data on the mechanisms of action regarding the relevance of *C. acnes* in the onset and development of acne, as well as the importance of the gut–skin axis, are becoming known. Classically, acne has been associated with an increase in androgens at the systemic and local levels, but the influence of IGF-1 and insulin, which in turn are dependent on diet, is transcendental. There is increasing evidence that the virulence and biofilm-forming characteristics of *C. acnes* strains present in acne lesions are more important than the mere presence of this bacteria. Likewise, the ability of *C. acnes* to generate proinflammatory lipids that aggravate the development of acne is also important. In addition, certain genetic polymorphisms have been found that are associated with the presence of acne, which could be related to greater severity or its development in adults.

Although the exact mechanisms by which the intestinal microbiota can influence the development and evolution of acne is unknown, studies in this regard have shown intestinal dysbiosis in these patients. The loss in SCCA-producing bacteria is remarkable, since these molecules have anti-inflammatory effects, and in acne, the inflammatory reaction that is established in the pilosebaceous unit is essential. A diet high in fat or rich in foods with a high glycemic index, which also affects the intestinal microbiota by increasing intestinal permeability, is also a factor that aggravates the development of acne. These data would indicate that a modulation of the intestinal microbiota could potentially influence the appearance and evolution of acne.

Diverse and varied in vitro studies have shown the potential beneficial effects of probiotics in this context; however, clinical trials with both topical and oral probiotics are scarce, although they have shown positive results. However, the evidence for potentially beneficial effects of oral probiotics is greater. Orally administered probiotics would exert their beneficial functions through the modulation of the intestinal microbiota, generating an anti-inflammatory response, restoring intestinal integrity, or through metabolic pathways involving IGF-1. Topical probiotics seem to produce their effects through the inhibition of growth of *C. acnes* in the pilosebaceous unit. Given the aggressiveness of some standard acne treatment, probiotics should continue to be investigated as an alternative or adjuvant therapy.

## Figures and Tables

**Table 1:** Main studies including gut microbiota data in patients with and acne vulgaris.

Reference	Methodology and Study Population	Key Results
Loveman 1955 [102].	A total of 10 patients with severe pustular acne and 10 healthy controls. Stool microbiological culture.	No significant differences were found in a small classic group of intestinal pathogenic bacteria compared to a group of 10 healthy controls
Volkova 2001 [103].	A total of 114 patients with acne. Stool microbiological culture.	54% of acne patients presented gut dysbiosis.
Deng 2018 [104].	A total of 43 treatment-naïve patients with different degrees of acne versus 43 age- and sex-matched healthy controls. NGS 16S rRNA.	Decreased alpha diversity in acne patients compared to healthy controls. No differences in severity. PCoA and ANOSIM analyses showed two different clusters for cases and controls. LefSe analysis found 38 differences between the groups of acne patients and healthy controls. Decrease in Firmicutes and increase in Bacteroidetes in the group of patients with acne. Decrease in Clostridial families such as Lachnospiraceae and Ruminococcaceae, producers of SCFA in patients with acne. PICRUSt analysis showed an increase in LPS synthesis pathways in acne patients.
Yan 2018 [108].	A total of 31 mild-moderate acne patients and 31 age- and sex-matched healthy controls. NGS 16S rRNA.	No differences were observed regarding alpha diversity between cases with acne and controls. PCA did not show different clusters. Gut microbiota between cases and controls was quite similar. Loss in *Bifidobacterium* and a slight gain in Proteobacteria in patients with acne.
Thompson 2020 [110].	Eight patients with acne before and after receiving minocycline as treatment and eight healthy age- and sex-matched controls. NGS 16S rRNA.	Not significant differences in alpha diversity were between acne patients before and after antibiotic therapy. Gut microbiota of patients with acne before taking antibiotics compared to healthy controls showed a decrease in *Lactobacillus iners*, *Lactobacillus zeae*, and *Bifidobacterium animalis*. After antibiotic treatment, gut microbiota of acne patients showed decreases in *Lactobacillus salivarius*, *Bifidobacterium adolescentis*, *Bifidobacterium pseudolongum*, *Bifidobacterium breve*, and *Akkermansia mucinophila* compared to healthy controls. Bacteroidetes increase in acne patients after treatment. Decrease in Firmicutes/Bacteroidetes ratio.
Rainer 2021 [111].	Eight subjects with acne after 4 weeks treatment with minocycline and eight age-, race-, and sex-matched healthy controls. NGS 16S rRNA.	Firmicutes/Bacteroidetes ratio was higher in controls and lower in patients with acne after treatment Slightly higher baseline Firmicutes/Bacteroidetes ratio was observed in patients with less-severe acne compared to those with greater severity.
Huang 2021 [105].	A total of 86 study subjects: 26 men with acne + 26 healthy control men and 17 women with acne + 17 healthy control women. NGS 16S rRNA and fecal metabolomic study.	Alpha diversity was lower in men with acne compared to control men. No significant differences were found between women with acne and control women. ANOSIM test showed four statistically significant clusters among the four study groups: Firmicutes and Bacteroidetes were significantly increased and decreased, respectively, in men with acne versus control men. These differences were not found in women with acne versus control women. At the genus level, decreases were observed in men with acne compared to control men from 18 genera. Many genera were SCFA producers. In women with acne compared to control women, an increase in *Clostridium sensu stricto* and a decrease in *Oscillibacter* and *Odoribacter* were observed. Men with acne tended to have impaired fatty acid metabolism while women with acne tended to have impaired amino acid metabolism.

PCoA: principal coordinates analysis, ANOSIM: analysis of similarities, LefSe: linear discriminant analysis effect size, SCFA: short-chain fatty acid, LPS: lipopolysaccharide, PCA: principal component analysis.

**Table 2:** Acne vulgaris and probiotics.

Reference	Study	Probiotic	Key Results
Bowe [112].	In vitro	*Streptococcus salivarius*	Bacteriocin inhibited *C. acnes* growth.
Oh [113].	In vitro	*Lactococcus* sp. HY 449	Bacteriocin inhibited *C. acnes* growth.
Deidda [114].	In vitro	*Lactobacillus salivarius* LS03	Bacteriocin inhibited *C. acnes* growth.
Lee [109].	In vitro	*Bifidobacterium adolescentis*	Antimicrobial activity against *C. acnes* and *Staphylococcus aureus.*
Wang [115].	In vitro	*Staphylococcus epidermidis*	Production of succinic acid through glycerol fermentation.
Cosseau [116].	In vitro	*Streptococcus salivarius* K12	Anti-inflammatory response; modulation of genes associated with epithelial adhesion.
Gueniche [117].	In vitro	*Lactobacillus paracasei* CNCM I-2126	Improvement of skin barrier function.
Al-Ghazzewi [120].	In vitro	*L. casei* NCFB 161, *L. acidophilus* NCFB 1748, *L. plantarum* DSM 12028, *L. gasseri* NCFB 2233, and *Lactococcus lactis* NCIMB 66 plus glucomannan hydrolysates of *Amorphophallus konjac*	Inhibition of *C. acnes* growth.
Lopes [121].	In vitro	Several *Bifidobacterium* and *Lactobacillus* strains	Adherence to keratin; inhibition of biofilm formation of pathogenic bacteria; limited ability to adhere to *C. acnes.*
Chae [122].	In vitro	*L. plantarum* APsulloc 331261 and APsulloc 331266	Inhibition of skin pathogen growth.
Espinoza-Monje [123].	In vitro and murine model	*Weissella viridescens* UCO_SMC3	Inhibition of *C. acnes* growth; anti-inflammatory effects.
Siver [124].	Clinical trial	*L. acidophilus and L. bulgaricus* (oral)	A total of 300 acne patients; 2 weeks of treatment. Clinical improvement in 80% of acne patients.
Jung [127].	RCT, open-label	*L. acidophilus* (5 × 10^9^ CFU/capsule), *L. bulgaricus* (5 × 10^9^ CFU/capsule) and *B. bifidum* (20 × 10^9^ CFU/capsule) (oral); two capsules/day	A total of 45 acne patients; three study groups (probiotic, minocycline, probiotic plus minocycline); 12 weeks of treatment. Patients treated with probiotic mixture plus minocycline had significantly better efficacy in terms of total number of lesions.
Fabbrocini [128].	RCT, double-blinded, placebo-controlled	Liquid supplement containing *Lactobacillus rhamnosus* SP1 at a dose of 3 × 10^9^ CFU/day (oral)	A total of 20 acne patients; 12 weeks of treatment. IGF-1 and FoxO1 gene expression in skin acne areas. Statistically significant reduction in the expression of the IGF-1 gene of 32% and a statistically significant increase in the FoxO1 gene of 65% in probiotic group. Clinical improvement in patients treated with probiotic.
Rahmayani [129]	Pre-experimental clinical study with a pretest/posttest	*B. lactis* W51, *B. lactis* W52, *L. acidophilus* W55, *L. casei* W56, *L. salivarius* W57, and *Lactococcus lactis* W58, with total bacterial cells > 10^8^ CFU per sachet (oral); two sachets/day	A total of 30 acne patients; 30 days of treatment. An increase in IL-10 was seen after probiotic mixture treatment.
Manzhalii [132].	RCT, controlled, nonblinded	*Escherichia coli* Nissle 1917 (oral); one capsule contained 2.5–25 × 10^9^ CFU; two capsules/day	A total of 82 patients with intestinal-borne dermatoses (some of them were diagnosed with acne); 1 month of treatment. Two study groups (patients treated with a conventional topical therapy consisting of ointments containing tetracycline, steroids, and retinoids; and patients treated with conventional topical therapy plus probiotic). A total of 89% of patients treated with *E. coli* Nissle 1917 improved significantly, while 56% improved in the group treated with only the conventional topical therapy.
Rinaldi [133].	RCT, double-blinded, placebo-controlled	*B. breve* BR03 (0.5 × 10^9^ CFU), *L. casei* LC03 (≥0.5 × 10^9^ CFU), and *L. salivarius* LS03 (≥1.0 × 10^9^ CFU) plus a botanical extract of *Solanum melongena* and *Echinacea* (oral); one sachet/day	A total of 114 acne patients. Four study groups (placebo, probiotics, botanical extracts, and probiotics plus botanical extracts); 8 weeks of treatment. A decreased number of acne lesions, rate of desquamation, rate of sebum secretion, and presence of *C. acnes* was found in patients treated with the probiotic mixture and botanical extract, and a mixture of both. Stronger effects were seen with the probiotic mix plus the botanical extract.
Kang [136].	RCT, double-blinded, placebo-controlled	Concentrated powder lotion obtained from supernatant culture of *Enterococcus faecalis* SL-5 (topical)	A total of 70 acne patients; 8 weeks of treatment. A decrease in inflammatory lesions was seen.
AOBiome [137].	RCT, double-blinded, placebo-controlled	*Nitrosomonas eutropha* (topical)	A total of 358 acne subjects; 12 weeks of treatment. A reduction in severity and a trend toward a reduction in inflammatory lesions was seen.
Sathikulpakdee [138].	RCT	*Lactobacillus paraceasei* MSMC 39-1 (topical)	A total of 104 acne patients; 4 weeks treatment. Topical probiotics vs. 2.5% benzoyl peroxide lotion were compared. Acne lesions and the erythema index were decreased.

CFU: colony-forming units; RCT: randomized clinical trial.

## Data Availability

Not applicable.
